# Efficacy of a behavioral self-help treatment with or without therapist guidance for co-morbid and primary insomnia -a randomized controlled trial

**DOI:** 10.1186/1471-244X-12-5

**Published:** 2012-01-22

**Authors:** Susanna Jernelöv, Mats Lekander, Kerstin Blom, Sara Rydh, Brjánn Ljótsson, John Axelsson, Viktor Kaldo

**Affiliations:** 1 Department of Clinical Neuroscience, Karolinska Institutet, SE-171 77 Stockholm, Sweden; 2 Osher Center for Integrative Medicine, Karolinska Institutet, SE-171 77 Stockholm, Sweden; 3 Stress Research Institute, Stockholm University, SE-106 91 Stockholm, Sweden

## Abstract

**Background:**

Cognitive behavioral therapy is treatment of choice for insomnia, but availability is scarce. Self-help can increase availability at low cost, but evidence for its efficacy is limited, especially for the typical insomnia patient with co-morbid problems. We hypothesized that a cognitive behaviorally based self-help book is effective to treat insomnia in individuals, also with co-morbid problems, and that the effect is enhanced by adding brief therapist telephone support.

**Methods:**

Volunteer sample; 133 media-recruited adults with insomnia. History of sleep difficulties (mean [SD]) 11.8 [12.0] years. 92.5% had co-morbid problems (e.g. allergy, pain, and depression). Parallel randomized (block-randomization, n ≥ 21) controlled "open label" trial; three groups-bibliotherapy with (n = 44) and without (n = 45) therapist support, and waiting list control (n = 44). Assessments before and after treatment, and at three-month follow-up. Intervention was six weeks of bibliotherapeutic self-help, with established cognitive behavioral methods including sleep restriction, stimulus control, and cognitive restructuring. Therapist support was a 15-minute structured telephone call scheduled weekly. Main outcome measures were sleep diary data, and the Insomnia Severity Index.

**Results:**

Intention-to-treat analyses of 133 participants showed significant improvements in both self-help groups from pre to post treatment compared to waiting list. For example, treatment with and without support gave shorter sleep onset latency (improvement minutes [95% Confidence Interval], 35.4 [24.2 to 46.6], and 20.6 [10.6 to 30.6] respectively), and support gave a higher remission rate (defined as ISI score below 8; 61.4%), than bibliotherapy alone (24.4%, p's < .001). Improvements were not seen in the control group (sleep onset latency 4.6 minutes shorter [-1.5 to 10.7], and remission rate 2.3%). Self-help groups maintained gains at three-month follow-up.

**Conclusions:**

Participants receiving self-help for insomnia benefited markedly. Self-help, especially if therapist-supported, has considerable potential to be as effective as individual treatment at lower cost, also for individuals with co-morbid problems.

**Trial Registration:**

ClinicalTrials.gov: NCT01105052

## Background

In the general population, about one third suffers from one or more symptoms of insomnia, and about 10% fulfill the criteria for a clinical diagnosis [1]. Insomnia entails substantial individual suffering, and costs to society [2] through factors such as drug-use, increases in risks for long-term sick-leave, major depression, and hypertension [3-6].

Despite the high prevalence and negative consequences of insomnia, only a small percentage is treated [7,8], most commonly with pharmacotherapy [8]. However, many individuals with insomnia would prefer non-pharmacological treatment if available [9,10]. Cognitive behavioral therapy (CBT) has been shown to be effective for insomnia [11-13], and is therefore considered treatment of choice [14,15]. Still, CBT is provided only to a minority, at least partially due to the limited availability of CBT therapists [16] and high initial costs [17].

To improve the availability of effective psychological treatments, self-help protocols have been developed for a number of problems, including anxiety, depression, and tinnitus [e.g. [18]]. Evaluations have often shown results comparable to face-to-face treatments [19], while self-help treatments are most likely more cost-effective [20,21]. Although thorough health-economic studies on the cost-effectiveness of self-help are still in their infancy, the potential for helping more people to a lower cost is obvious. Self-help protocols have been developed also for insomnia, and in a recent meta-analysis, Van Straten & Cuijpers concluded they are effective for individuals with primary insomnia [17].

However, the majority of patients with insomnia present with a range of co-morbid problems [22]. In earlier studies, these patients were often excluded, since other states such as psychiatric conditions and medical problems including other sleep disorders were considered to cause the insomnia.

This exclusion reduces the generalizability of the empirical support for CBT for insomnia since insomnia only rarely occurs without co-morbid conditions [23]. For example, in one study where insomnia diagnoses were assessed, only 20% of the participants were diagnosed with primary insomnia, while 52% were diagnosed with insomnia secondary to a mental disorder (44%) or to a medical condition or substance abuse (8%) [24].

The causal direction of the different conditions is often very difficult to establish, possibly due to bi-directional relationships between insomnia and other disorders. Hence, it has been suggested that the term co-morbid (rather than secondary) insomnia be used and that it may not be necessary to treat the "other" disorder first [25]. Indeed, in a recent RCT, Edinger et al [26] showed that CBT for insomnia delivered individually is useful also for patients with co-morbidities, and a relatively recent review concludes that CBT for insomnia (delivered individually or as group treatments) is promising for individuals with medical and psychiatric co-morbidity [27]. By contrast, data is very limited on self-help treatments for this broader population of insomnia sufferers. It is therefore crucial to find out if the previous positive results shown for individuals with primary insomnia without co-morbidities, also generalize to the larger group of patients including those with co-morbidities.

Therapist guidance can improve the effects of self-help treatments and reduce drop-out rates [21,28]. This seems true also for self-help CBT for insomnia [17], although the few studies comparing self-help with and without therapist guidance are limited by rather low power and have excluded individuals with co-morbidities [29,30]. It is also unclear whether the differences between guided and unguided participants seen directly after treatment are stable over time. In addition, previous evaluations of insomnia treatments have focused mainly on sleep-timing measures, while studies investigating effects of insomnia treatments on daytime functioning and psychological distress are lacking.

The objective of the present study was to compare the effects of a CBT-based self-help treatment for insomnia, given with or without therapist support, to a waiting list control group. To compensate for limitations in previous studies we wanted to study individuals from the general population, including patients with co-morbidities, and to also evaluate day time functioning and psychological distress.

Our hypotheses were that participants would benefit from treatment both directly after the treatment and at three-month follow up, and that support would enhance outcome. We hypothesized that these improvements would be seen in response and remission rates, sleep timing, subjective measures of sleep, and daytime functioning. No differences in outcome due to co-morbid problems were expected.

## Methods

### Design and Randomization

This study was a randomized controlled trial with three arms. One-hundred and thirty three participants were block-randomized (smallest block n = 21) by KB and SR via a true randomization process http://www.random.org to bibliotherapy with support (n = 44), to bibliotherapy only (n = 45), or to the waiting list control group (n = 44). Self report measures precluded blinding.

### Ethics

This study was approved by the Regional Ethical Review Board in Stockholm on February 2, 2008, identification number 2008/23-31/4.

### Participants

Participants were recruited from all over Sweden, through media and websites. Interested individuals were directed to a web-site with a description of the study, informed consent and screening forms. All assessments were conducted on the Internet or telephone. No monetary compensation was given for participation, but the participants did not pay for the treatment.

Inclusion criteria were: be at least 18 years old; meet research diagnostic criteria (RDC) for insomnia according to the American Academy of Sleep Medicine [31]; have insomnia at a clinical level defined as more than 10 points on the Insomnia Severity Index (ISI) [32]; duration of insomnia more than four weeks, have adequate Swedish language skills; have access to a computer and Internet for filling out online forms; be available for assessments and treatment during the course of the study; absence of other sleep disorders that require other treatment (e.g. sleep apnea); no severe somatic or psychiatric disorder that temporally precedes insomnia onset and from the patient's point of view is the only cause of insomnia, contraindicative of the treatment (e.g. bipolar disorder), or require other treatment that cannot be combined with this insomnia treatment; no severe depression (defined as > 30 points on MADRS-S, see below) or suicidality (≥ 4 points on MADRS-S, item 9); no severe alcohol or drug dependence; no night shift work; no ongoing other insomnia treatment or previous CBT for insomnia; not having a self-help book for insomnia based on CBT techniques at home. Individuals were not excluded due to using sleep medications.

The inclusion criteria were assessed from screening forms and telephone interviews. If diagnoses or cut-offs were ambiguous, they were discussed in clinical supervision (SJ), and if needed, complementary data was gathered through an additional telephone interview.

### Co-morbid insomnia

In the present study, insomnia diagnosis was assigned following a structured interview based on the clinical interview developed by Morin & Espie [33], translated into Swedish, and with the addition of RDC for insomnia [31]. The interview also included questions regarding the other inclusion criteria (see above), e.g. current and previous medical, psychiatric and psychological diagnoses and problems, and sleep medication use. One section also included individually tailored questions for clarifying any uncertainties from the screening forms. Based on these interviews, interviewees were assigned a diagnosis of primary insomnia (PI) or co-morbid insomnia (CMI). Those diagnosed with PI met RDC for primary insomnia and had no findings on the structured interview suggesting a medical, psychiatric or medication cause for their insomnia. Participants assigned a CMI diagnosis met RDC criteria for insomnia disorder and had findings on the structured interview suggesting their insomnia be at least partially resulting from or affected by a concurrent active psychiatric or medical problem, insomnia was a central complaint and was not attributed exclusively to the other disorder. Patients who met RDC critera for insomnia and had a co-morbid condition, but where the insomnia was not the primary complaint and was attributed exclusively to the other disorder (be it sleep, medical, or psychiatric) were excluded from this study.

The screening for sleep apnea received special attention, since apnea frequency and/or severity can increase as a result of the sleep deprivation often following the so called sleep restriction (or sleep compression) used during CBT-treatment of insomnia. The ESS scale (see below) and two structured questions on snoring and apneas were used in the interview. When indicative of sleep apnea, follow-up questions were made and individuals with plausible or diagnosed sleep apnea were excluded and, if not already in treatment, referred to a sleep medicine clinic.

### Intervention

The self-help book [34] is based on well-established CBT-models and techniques [32,35], and has two parts. The first part consists of psycho education about sleep and insomnia, including a simplified model for insomnia and three exemplary cases. The second part presents techniques, e.g. relaxation and visualization techniques, sleep restriction and stimulus control, cognitive restructuring techniques, and sleep hygiene. The techniques are presented under headings such as Stress-Relief, Work on Thoughts, and Help Your Sleep-Rhythm. One section aims to analyze the reader's personal situation using a Sleep Diary and a so called Treatment Guide designed to help determining which techniques to focus on. The treatment guide primarily suggests using the techniques sleep restriction and stimulus control. Sleep restriction instructions include setting a sleep window to number of hours slept during the previous 4-7 nights based on sleep diary recordings (no lowest limit given), fixating the wake-up time, and increasing the scheduled time in bed with 15 minutes contingent on sleep efficiency exceeding 85% during the last 4-7 days. A separate chapter focuses on sleep medication and sleep medication tapering. A CD with audio relaxation and visualization exercises is included. The final chapter focuses on relapse prevention.

#### Treatment phase

The book was sent to the participants in the two treatment groups, together with a letter encouraging them to start reading and working with the program as soon as possible. Participants in the group without therapist support then worked independently during the six week treatment period.

Participants in the group receiving therapist support were contacted by telephone to schedule an appointment for each week of the treatment (i.e. six telephone appointments), and were encouraged to start reading the book, filling out the digital Treatment Guide and Sleep Diary and e-mailing these to the therapist before their first scheduled telephone appointment.

All telephone appointments were conducted using a structured guide. For instance, the first appointments focused on getting started, analyzing the patients' situation, and setting a sleep window for sleep restriction, and the final telephone appointment focused on relapse prevention. Each appointment was kept to a maximum of 15 minutes during which therapists also coded progress and homework assignments on a structured evaluation sheet.

Therapists in the present study were in their final year of training as clinical psychologists. Adherence to the treatment protocol was ascertained through the use of the written therapist guide, the structured evaluation sheet, and the self-help manual and supervision of therapists by an experienced clinician (SJ).

Participants in the control condition received the treatment book without support after the three-month follow-up assessment.

### Measures

All self-report questionnaires were filled out over the Internet, which improves the quality of data since missing items are not accepted and type of input can be automatically validated before data is submitted by the participants. In addition, the relative anonymity of questionnaires on the Internet has been suggested to reduce social desirability of respondents [36].

#### Primary outcome measures

*The Insomnia Severity Index *(ISI) [32] is a much used, 7-item patient-reported outcome measure assessing the severity of initial, middle and late insomnia; sleep satisfaction; interference of insomnia with daytime functioning; noticeability of sleep problems by others; and distress about sleep difficulties. A 5-point scale (0-4) is used to rate each item, yielding a total score of 0 to 28. The ISI has adequate psychometric properties and is sensitive to measuring treatment response [37]. Treatment response and remission rates were calculated from the ISI; as suggested by Morin et al [38], participants were considered treatment responders if their ISI score changed with 8 points or more compared to pre-assessment, and as treatment remitters if their absolute ISI score was less than 8.

*Sleep timing *was measured with *a sleep diary *[32], the most widely used outcome measure in insomnia research [39], and was recorded during one week at each assessment point. The sleep diary includes registration of bed time, time of falling asleep, length of night time awakenings, time of waking up and time of getting out of bed. Means of the daily ratings were calculated for sleep onset latency, wake after sleep onset, total sleep time, and sleep efficiency.

#### Secondary outcome measures

*The sleep diary *was also used to gauge subjective sleep quality, stress at bed time, and overall day-time functioning. The latter included questions for day-time fatigue ("how tired you have felt today"), and positive day-time ratings ("how alert/well functioning/happy you have felt today"). Each of these items were rated from 0 'not at all' to 5 'very much so' and the three positive day-ratings were combined to a composite score (Crohnbach's alpha = 0.893). All subjective sleep measures and measures of day-time functioning were calculated as means over the week.

*The Dysfunctional Beliefs and Attitudes about Sleep *(DBAS) is a 30-item self-report measure identifying sleep disruptive cognitions [40]. Although developed as a visual analogue scale, it was transformed into a Likert-type scale with responses 0-10 for the use on a web-site. Scores range is 0-300.

*The Sleep-Related Behaviour Questionnaire *(SRBQ) is used to assess counter productive safety behaviors in insomnia [41]. The scale has 32 items which are scored between 1 (almost never) to 5 (almost always), yielding a total score range of 32-160.

*The Perceived Stress Scale-10 items *(PSS-10) measures the perceived stress in daily life [42,43]. The PSS-10 has 10 items with response alternatives 0 (never) to 4 (very often). Total score ranges from 0-40.

*The Clinical Outcomes in Routine Evaluation-Outcome Measure *(CORE-OM) evaluates general psychological distress [44]. The 28-item scale used has response alternatives 0 (often) to 4 (almost all the time), yielding a total score between 0 and 112.

### Power calculation and sample size

Power estimates based on the effects found in previous studies (e.g. [29], d = 0.6 to 1.0) suggested at least 44 participants in each group, for a power of 80%.

### Statistical methods

One way ANOVAs and χ^2 ^associations were used to compare groups on background variables. To reduce the risk of mass-significance due to the many outcome measures, repeated measures MANOVAs were initially conducted with treatment group as between-subjects variable. For the MANOVAs, the outcome variables were combined into three conceptually coherent groups; sleep timing, subjective sleep, and measures of day-time functioning and psychological distress. Three MANOVAs were used for the pre- to post-comparison, and three MANOVAs were used to compare pre- to follow-up assessments. When a MANOVA showed significant interaction, follow-up tests were performed with a 2 × 2 ANOVA for each separate outcome measure.

Due to significant pre-treatment differences in Total Sleep Time (F_(2,129) _= 6.46, p = .002), DBAS (F_(2,130) _= 3.47, p = .034) and SRBQ (F_(2,130) _= 3.36, p = .038) η_p_^2^-values (Eta squared) were used to calculate between-groups effect sizes for interactions, rather than using Cohen's d for between group differences. To estimate within group changes, Cohen's d was used. To evaluate effects of the general burden of co-morbidity on outcome, number of co-morbid problems was correlated (Spearman's Rho (ρ) with change-scores for the ISI. To test for the impact of specific co-morbid problems, repeated measures ANOVAs were calculated with presence or non-presence of each of the most prevalent problems as between-groups-factor, and with ISI as the dependent variable at the three different time-points.

For all analyses of variance, Huynh-Feldt corrections were applied when sphericity could not be assumed, based on Mauchly's Test of Sphericity. Thirteen outliers were found in sleep diary data, and following the recommendations by Tabachnick & Fidell [45], score alteration was performed in order not to lose valuable data, but lessen the impact of outliers. Analyses were conducted using PASW statistics 17 and 18 (SPSS Inc. Chicaco, Illinois).

## Results

### Attrition, drop-out, adherence and use of other treatments

**Figure 1:**
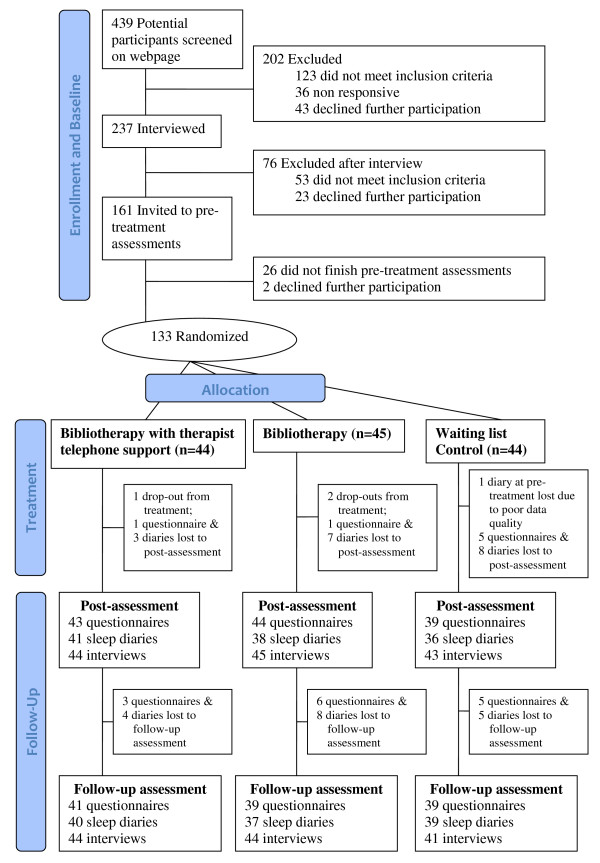
**CONSORT Flow chart**. Participant flow throughout the study.

Attrition and drop-out at post- and follow-up assessments were low (see Figure 1).

To measure adherence, participants in the treatment groups estimated how many hours per week they had spent on treatment and how much of the book they had read during treatment. Participants in the treatment group with support reported having read on average 84% (SD = 14%) of the book, and those in the group without support 77% (SD = 27%). Corresponding figures for time spent on treatment were 6.8 (SD = 5.9) and 7.3 (SD = 12.1) hours per week. There were no significant differences between the groups on these adherence measures.

In the waiting list group, six participants sought other treatments during the first period (from pre- to post-assessments), and two during the second period (from post-assessment to follow-up). In the group receiving bibliotherapy with therapist support, only one participant sought another treatment during the first period, and two during the second. In the bibliotherapy group the corresponding figures were two and six participants respectively. A trend for an association between number of participants seeking other treatments, and group, is seen for the first period (χ^2 ^= 5.174, df = 2, p = .075, N = 131), but not for the second. Data from these individuals is also included in the analyses.

### Recruitment and baseline data

Table 1 presents baseline data of the 133 participants from all over Sweden. Only 7.5% of included participants were diagnosed with primary insomnia with no concurrent active disorder or problem affecting sleep. Participants reported on average 3.4 (SD = 2.3) co-morbid problems, the most common diagnoses being allergic diseases (57.9%), acute (31.6%) and chronic (15%) pain, stress (29.3%), restless legs (25.6%), nightmares (24.8%), snoring (23.3%), bruxism (21.8%), high blood pressure (15%), nocturia (11.3%), tinnitus (11.3%), depression (11.3%), and anxiety (10.5%). Using cut-off scores on MADRS-S, 30.3% of participants suffered from mild depression (MADRS-S 13 -19), and 23.3% would have been diagnosed with major depressive disorder (MADRS-S 20-30). Neither the number of co-morbid problems or presence of any specific problem, nor any of the background variables, differed between the three groups (see below). However, sleep medication use was significantly higher in the waiting list control group compared to the group receiving bibliotherapy without support.

**Table 1:** Baseline Characteristics of Participants

Characteristic	Bibliotherapy with support (n = 44)	Bibliotherapy (n = 45)	Waiting list control (n = 44)	Total (N = 133)	*P *Value^a^
Age, mean (SD), years	50.8 (11.8)	47.4 (13.3)	45.4 (16.0)	47.9 (13.9)	.18
Women, No. (%)	33 (75)	36 (80)	40 (90.9)	109 (82)	.14
Marital status, No. (%)					
Married/living with partner	30 (68.1)	37 (82.2)	29 (65.9)	96 (72.2)	.64
Separated/Divorced/Widowed	6 (13.6)	2 (4.4)	5 (11.4)	13 (9.8)	
Singel/Other	8 (18.2)	6 (13.3)	10 (22.7)	24 (18.0)	
Educational level, No. (%)					
Compulsary school/Other	2 (4.5)	8 (17.8)	3 (6.8)	13 (9.8)	.30
Upper secondary school	13 (29.5)	16 (35.6)	18 (40.9)	47 (35.3)	
College/University	29 (65.9)	21 (46.7)	23 (52.3)	73 (54.9)	
Current occuptaion ^b^, No.(%)					
Work/Full time studies	27 (61.4)	30 (66.7)	25 (56.8)	82 (61.7)	.63
On sick-leave	9 (20.4)	5 (11.1)	4 (9.1)	18 (13.5)	.25
Unemployed	3 (6.8)	2 (4.4)	2 (4.5)	7 (5.3)	.98
Retired	6 (13.6)	8 (17.8)	9 (20.5)	23 (17.3)	.70
Works at home/On parental leave/Other	9 (20.4)	5 (11.1)	6 (13.3)	20 (15.0)	.44
Economic situation, No. (%)					
Bad/Very bad	3 (6.8)	3 (6.7)	7 (15.9)	13 (9.8)	.36
Neither good nor bad	13 (29.5)	8 (17.8)	14 (31.8)	35 (26.3)	
Good/Very good	28 (63.6)	34 (75.6)	23 (52.3)	85 (63.9)	
Depression level (MADRS) at screening, mean (SD)	14.1 (6.38)	12.8 (6.05)	15.2 (5.85)	14.0 (6.13)	.19
Insomnia Severity (ISI) at screening, mean (SD)	18.2 (3.5)	18.3 (3.3)	18.3 (3.3)	18.3 (3.3)	.99
History of Insomnia, mean (SD), years	11.7 (13.1)	12.7 (13.1)	11.1 (9.4)	11.8 (12.0)	.82
Co-morbid Insomnia No. (%)	39 (88.6)	44 (97.8)	40 (90.9)	123 (92.5)	.45
Number of co-morbid problems, mean (SD)	2.8 (2.3)	3.7 (2.2)	3.5 (2.2)	3.4 (2.3)	.17
Sleep medication use at pre-assessment, No. (%)	20 (45.5)	14 (31.1)	25 (56.8)	59 (44.3)	.05

^a ^*P *values are based on analysis of variance or _*X*_^2 ^tests

^b ^more than 1 choice possible

Participants were enrolled and screened between February 26^th ^and March 30^th ^2008. Pre-treatment assessments were conducted between March 27^th ^and April 28^th^, post-treatment assessments between May 22^nd ^and June 13^th^, and follow-up between September 11^th ^and October 27^th^, all in 2008.

### Numbers analyzed

Except correlations and ANOVAS analyzing effects of co-morbidity, all outcome analyses are computed for intent-to-treat data with last observation carried forward, based on 133 pre-assessment questionnaires and 132 pre-assessment sleep diaries (one in the control group lost due to poor data quality).

### Primary outcomes

#### Treatment response and remission rates

**Figure 2:**
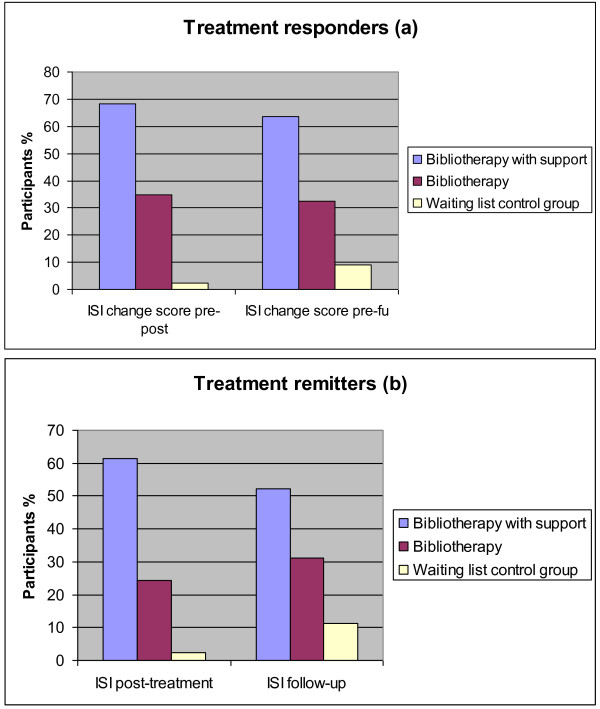
**Proportion of treatment responders and remitters according to treatment group**. (a) Treatment response defined as a change score on the Insomnia Severity Index of 8 points or more from pre-treatment. (b) Treatment remission defined as an Insomnia Severity Index score of less than 8 points.

As seen in Figure 2, there were very few responders and remitters in the waiting list control group (1 (2.27%) for both variables), more in the bibliotherapy group (15 (34.9%) and 11 (24.4%)), but by far the most responders and remitters were seen in the group receiving bibliotherapy with therapist support (30 (68.2%) and 27 (61.4%)) at post-assessment. The differences between groups remained at three-month follow-up, and were significant both for post- and three-month follow-up assessments (χ^2 ^= 17.047-42.289, df = 2, all p's < .001, N = 133).

#### Sleep timing

Two MANOVAs were conducted with sleep diary data concerning aspects of sleep timing (i.e. sleep onset latency, wake after sleep onset, total sleep time, and sleep efficiency) as dependent variables, the first comparing all three groups between pre- and post-treatment, and the second between pre-treatment and three-month follow-up. Both MANOVAs showed significant interactions (pre-post: F_(8, 252) _= 9.351, *p *= .000, η_p_^2 ^= 0.229; pre-follow up: F_(8, 252) _= 3.785, *p *= .000, η_p_^2 ^= 0.107).

Separate analyses were performed to establish which interactions were significant (see tables 2 and 3). From pre- to post-assessments, bibliotherapy with therapist support gave larger gains than did waiting in all sleep timing measures except total sleep time, and also larger gains in wake after sleep onset, and sleep efficiency, compared to bibliotherapy. Bibliotherapy improved sleep onset latency and sleep efficiency more than did waiting. Regarding changes from pre- to three-month follow-up assessments, bibliotherapy with therapist support now produced larger gains in all sleep timing measures, compared to waiting. However, only the difference in sleep efficiency remained between bibliotherapy with and without support. Finally, differences between bibliotherapy and waiting now included total sleep time, as well as the earlier differences seen in sleep onset latency and sleep efficiency.

**Table 2:** Sleep timing measures, Mean (SD), Change values and Effect Sizes

	Pre	Post	Fu3	Change Pre to Post		Change Pre to Fu3	
	Mean (SD)	Mean (SD)	Mean (SD)	Mean (95% CI)	Effect size (*d*)^a^	Mean (95% CI)	Effect size (*d*)
Sleep Onset Latency, minutes							
Bibliotherapy with support (n = 44)	60.3 (41.0)	24.9 (17.0)	33.5 (22.6)	-35.4 (-46.6 to -24.2)	-1.13	-26.8 (-39.0 to -14.5)	-0.81
Bibliotherapy (n = 45)	65.7 (45.1)	45.1 (32.2)	43.0 (28.4)	-20.6 (-30.6 to -10.6)	-0.53	-22.7 (-34.8 to -10.5)	-0.60
Wait-list control (n = 43)	66.9 (42.1)	62.2 (42.4)	62.4 (45.1)	-4.6 (-10.7 to 1.5)	-0.11	-4.5 (-12.0 to 3.0)	-0.10
Wake After Sleep Onset, minutes							
Bibliotherapy with support (n = 44)	53.6 (40.4)	17.0 (16.8)	26.8 (29.3)	-36.5 (-47.7 to -25.4)	-1.18	-26.8 (-38.9 to -14.6)	-0.76
Bibliotherapy (n = 45)	47.5 (33.6)	32.0 (27.0)	30.2 (28.7)	-15.6 (-24.6 to -6.5)	-0.51	-17.4 (-26.6 to -8.2)	-0.55
Wait-list control (n = 43)	36.9 (29.5)	30.4 (28.9)	25.3 (25.0)	-6.5 (-12.5 to -0.4)	-0.22	-11.6 (-19.4 to -3.7)	-0.42
Total Sleep Time, hours							
Bibliotherapy with support (n = 44)	5.56 (1.44)	6.07 (1.02)	6.52 (0.95)	0.51 (0.20 to 0.83)	0.41	0.97 (0.56 to 1.37)	0.79
Bibliotherapy (n = 45)	5.73 (0.89)	6.22 (1.02)	6.46 (0.96)	0.49 (0.26 to 0.73)	0.51	0.73 (0.46 to 1.00)	0.79
Wait-list control (n = 43)	6.40 (1.09)	6.55 (1.26)	6.74 (1.11)	0.14 (-0.14 to 0.42)	0.13	0.34 (0.09 to 0.59)	0.31
Sleep Efficiency, %							
Bibliotherapy with support (n = 44)	67.1 (15.5)	85.4 (6.8)	83.3 (8.3)	18.3 (14.4 to 22.2)	1.53	16.2 (11.7 to 20.6)	1.30
Bibliotherapy (n = 45)	68.3 (11.1)	76.4 (10.9)	78.4 (9.8)	8.1 (5.8 to 10.3)	0.74	10.1 (7.6 to 12.6)	0.96
Wait-list control (n = 43)	71.5 (11.2)	74.5 (11.5)	76.1 (10.5)	3.0 (0.5 to 5.4)	0.27	4.5 (1.8 to 7.2)	0.42

Abbreviations: Pre, Pre-treatment assessment; Post, Post-treatment assessment; Fu3, 3-month follow-up assessment

^a ^Effect sizes calculated within groups, pre-post, and pre-fu3, with pooled SDs. (A negative effect size represents a decrease in the measure, a positive effect size an increase.)

**Table 3:** Sleep timing measures, interactions between groups

	Pre to Post						Pre to Fu3					
	BTS v/s WL		BTS v/s BT		BT v/s WL		BTS v/s WL		BTS v/s BT		BT v/s WL	
	*p*	Effect size (η_p_^2^)^a^	*p*	Effect size (η_p_^2^)	*p*	Effect size (η_p_^2^)	*p*	Effect size (η_p_^2^)	*p*	Effect size (η_p_^2^)	*p*	Effect size (η_p_^2^)
SOL	< .001	0.21	.06	0.04	.01	0.08	.003	0.10	.64	0.00	.02	0.07
WASO	< .001	0.20	.005	0.09	.11	0.03	.04	0.05	.23	0.02	.35	0.01
TST	.09	0.03	.92	0.00	.06	0.04	.01	0.07	.34	0.01	.04	0.05
SE	< .001	0.33	< .001	0.19	.004	0.09	< .001	0.19	.02	0.06	.004	0.09

Abbreviations: Pre, Pre-treatment assessment; Post, Post-treatment assessment; Fu3, 3-month follow-up assessment; BTS, Bibliotherapy with support; WL, Waiting list control group; BT, Bibliotherapy; SOL, Sleep Onset Latency; WASO, Wake After Sleep Onset; TST, Total Sleep Time; SE, Sleep Efficiency

^a ^η_p_^2 ^(Eta 2) is interpreted as explained variance in the sample

### Ancillary analyses

#### Subjective sleep measures, insomnia severity and sleep quality

Two MANOVAs were conducted with questionnaire ratings of subjective sleep as dependent variables (i.e. insomnia severity (ISI), sleep related behaviors (SRBQ), and dysfunctional beliefs (DBAS), and sleep diary data concerning subjective measures of sleep; bed time stress level and sleep quality). Both MANOVAs showed statistically significant effects of interaction (pre-post: F_(10, 250) _= 11.358, p < .001, η_p_^2 ^= .312; pre-follow up: F_(8, 252) _= 7.174, p < .001, η_p_^2 ^= .185).

Separate 2 × 2 ANOVAs were performed to establish which interactions were significant (see tables 4 and 5). From pre- to post-assessments, bibliotherapy with therapist support produced larger improvements in all subjective sleep ratings than did waiting, and also larger improvements than bibliotherapy alone. Bibliotherapy (without therapist support) resulted in larger improvements than did waiting, in all aspects but bed time stress levels. At three-month follow-up, all changes were maintained in the two treatment groups, and the differences between groups remained stable.

**Table 4:** Subjective sleep measures; Mean (SD), Change values and Effect Sizes

	Pre	Post	Fu3	Change Pre to Post		Change Pre to Fu3	
	Mean (SD)	Mean (SD)	Mean (SD)	Mean (95% CI)	Effect size (*d*)^a ^	Mean (95% CI)	Effect size (*d*)
Insomnia Severity Index							
Bibliotherapy with support (n = 44)	17.5 (3.5)	7.6 (4.5)	8.4 (5.1)	-9.9 (-11.3 to -8.5)	-2.46	-9.1 (-10.7 to -7.5)	-2.08
Bibliotherapy (n = 45)	16.6 (3.8)	11.2 (4.2)	10.4 (4.8)	-5.3 (-6.6 to -4.0)	-1.35	-6.2 (-7.5 to -4.8)	-1.43
Waiting list control group (n = 44)	16.7 (3.7)	15.6 (4.7)	14.2 (4.7)	-1.1 (-2.2 to 0.0)	-0.26	-2.5 (-3.5 to -1.5)	-0.59
Sleep Related Behaviours Questionnaire							
Bibliotherapy with support (n = 44)	83.6 (14.5)	57.8 (16.4)	58.5 (16.0)	-25.8 (-30.4 to -21.2)	-1.67	-25.1 (-29.8 to -20.8)	-1.64
Bibliotherapy (n = 45)	77.1 (12.8)	66.6 (16.0)	61.5 (17.5)	-10.5 (-14.1 to -6.9)	-0.72	-15.6 (-20.5 to -10.7)	-1.02
Waiting list control group (n = 44)	83.4 (12.8)	79.5 (13.8)	76.9 (13.6)	-3.9 (-6.9 to -0.9)	-0.29	-6.5 (-9.4 to -3.5)	-0.49
Dysfunctional Beliefs and Attitudes about Sleep							
Bibliotherapy with support (n = 44)	125.2 (31.3)	58.0 (30.8)	-*	-67.2 (-77.0 to -57.4)	-2.17	- *	- *
Bibliotherapy (n = 45)	116.0 (31.8)	85.8 (37.0)	-	-30.1 (-41.4 to -18.9)	-0.88	-	-
Waiting list control group (n = 44)	133.5 (31.1)	122.7 (30.7)	-	-10.8 (-16.2 to -5.4)	-0.35	-	-
Bed Time Stress ^b^							
Bibliotherapy with support (n = 44)	1.5 (1.1)	0.49 (0.74)	0.55 (0.88)	-1.03 (-1.34 to -0.72)	-1.08	-0.97 (-1.29 to -0.64)	-0.95
Bibliotherapy (n = 45)	0.80 (0.92)	0.68 (0.85)	0.52 (0.74)	-0.12 (-0.34 to 0.10)	-0.14	-0.28 (-0.51 to -0.06)	-0.36
Waiting list control group (n = 43)	1.2 (1.1)	1.1 (1.1)	1.1 (1.1)	-0.05 (-0.33 to 0.22)	-0.09	-0.08 (-0.33 to 0.16)	-0.09
Sleep Quality ^c^							
Bibliotherapy with support (n = 44)	2.6 (0.66)	3.6 (0.65)	3.5 (0.71)	1.0 (0.75 to 1.2)	1.53	0.93 (0.68 to 1.2)	1.31
Bibliotherapy (n = 45)	2.6 (0.53)	3.1 (0.57)	3.1 (0.65)	0.57 (0.39 to 0.75)	0.91	0.53 (0.32 to 0.75)	0.84
Waiting list control group (n = 43)	2.9 (0.56)	3.0 (0.66)	3.1 (0.61)	0.11 (-0.07 to 0.28)	0.16	0.20 (0.03 to 0.36)	0.34

Abbreviations: Pre, Pre-treatment assessment; Post, Post-treatment assessment; Fu3, 3-month follow-up assessment,

^a ^Effect sizes calculated within groups, pre-post, and pre-fu3, with pooled SDs. (A negative effect size represents a decrease in the measure, a positive effect size an increase.)

^b ^Scored on a scale 0-5, with 0 indicating no stress at all, and 5 indicating very high stress

^c ^Scored on a scale 1-5, with 1 indicating very poor sleep quality, and 5 indicating very good sleep quality

* DBAS-data missing from 3-month follow-up

**Table 5:** Subjective sleep measures; Interactions

	Pre to Post						Pre to Fu3					
	BTS v/s WL		BTS v/s BT		BT v/s WL		BTS v/s WL		BTS v/s BT		BT v/s WL	
	*p*	Effect size (η_p_^2^)^a^	*p*	Effect size (η_p_^2^)	*p*	Effect size (η_p_^2^)	*p*	Effect size (η_p_^2^)	*p*	Effect size (η_p_^2^)	*p*	Effect size (η_p_^2^)
ISI	< .001	0.52	< .001	0.20	< .001	0.21	< .001	0.36	.006	0.08	< .001	0.18
SRBQ	< .001	0.42	< .001	0.23	.007	0.08	< .001	0.33	.008	0.08	< .002	0.10
DBAS	< .001	0.53	< .001	0.21	.003	0.10	-*	-	-	-	-	-
Bed Time Stress	< .001	0.20	< .001	0.20	.71	0.00	< .001	0.18	.001	0.12	.23	0.02
Sleep Quality	< .001	0.27	.008	0.08	< .001	0.13	< .001	0.21	.02	0.06	.02	0.06

Abbreviations: Abbreviations: Pre, Pre-treatment assessment; Post, Post-treatment assessment; Fu3, 3-month follow-up assessment; BTS, Bibliotherapy with support; WL, Waiting list control group; BT, Bibliotherapy; ISI, Insomnia Severity Index; SRBQ, Sleep Related Behaviours Questionnaire; DBAS, Dysfunctional Beliefs and Attitudes about Sleep

^a ^η_p_^2 ^(Eta-2) is interpreted as explained variance in the sample

* DBAS-data missing from 3-month follow-up

#### Day-time functioning and psychological distress

Two MANOVAs were conducted with diary ratings of day-time functioning (i.e. Positive Day Time Ratings and Day Time Fatigue), and questionnaire ratings of perceived stress (PSS) and psychological distress (CORE-OM) as dependent variables. Both MANOVAs showed statistically significant effects of interaction (pre-post: F_(8, 252) _= 3.724, p < .001, η_p_^2 ^= .106; pre-follow up: F_(8, 252) _= 2.193, p = .029, η_p_^2 ^= .065).

Again, separate 2 × 2 analyses were performed to establish which interactions were significant (see tables 6 and 7). From pre- to post-assessments, bibliotherapy with therapist support produced larger improvements on all measures of day-time functioning and psychological distress than did waiting, and also larger improvements than bibliotherapy alone in all aspects but perceived stress. Bibliotherapy alone did not result in larger improvements in these measures compared to waiting. At three-month follow-up the differences between bibliotherapy with support and waiting remained stable, with the exception of psychological distress. The bibliotherapy groups, with or without support, now had similar improvements in all measures. At this assessment point, participants who had received bibliotherapy without support had improved more on day-time ratings and fatigue than participants in the waiting list control condition.

**Table 6:** Day time functioning and psychological distress; Mean (SD), Change values and Effect sizes

	Pre	Post	Fu3	Change Pre-Post		Change Pre-Fu3	
	Mean (SD)	Mean (SD)	Mean (SD)	Mean (95% CI)	Effect size (*d*)^a^	Mean (95% CI)	Effect size (*d*) ^a^
Positive Day Time Ratings							
Bibliotherapy with support (n = 44)	2.5 (0.79)	3.2 (0.70)	3.1 (0.74)	0.69 (0.46 to 0.92)	0.94	0.58 (0.34 to 0.82)	0.78
Bibliotherapy (n = 45)	2.6 (0.65)	3.0 (0.72)	3.0 (0.84)	0.38 (0.19 to 0.58)	0.58	0.44 (0.21 to 0.67)	0.58
Waiting list control (n = 43)	2.6 (0.73)	2.8 (0.75)	2.7 (0.76)	0.17 (-0.02 to 0.36)	0.27	0.13 (-0.07 to 0.32)	0.13
Day Time Fatigue (DTF)							
Bibliotherapy with support (n = 44)	2.8 (0.98)	1.5 (1.0)	1.8 (1.1)	-1.23 (-1.54 to -0.91)	-1.31	-0.98 (-1.3 to -0.66)	-0.96
Bibliotherapy (n = 45)	2.7 (0.82)	2.2 (0.99)	2.1 (0.94)	-0.49 (-0.81 to -0.17)	-0.55	-0.61 (-0.92 to -0.31)	-0.68
Waiting list control (n = 43)	2.7 (0.82)	2.4 (0.89)	2.6 (1.0)	-0.28 (-0.50 to -0.05)	-0.35	-0.14 (-0.42 to 0.13)	-0.11
Perceived Stress Scale (PSS)							
Bibliotherapy with support (n = 44)	17.4 (7.4)	13.1 (5.7)	13.2 (5.9)	-4.3 (-6.0 to -2.6)	-0.65	-4.2 (-6.1 to -2.3)	-0.63
Bibliotherapy (n = 45)	18.8 (6.2)	16.1 (7.1)	15.1 (7.4)	-2.7 (-4.2 to -1.1)	-0.41	-3.6 (-5.2 to -2.0)	-0.54
Waiting list control (n = 44)	19.0 (6.8)	17.2 (6.3)	17.3 (7.2)	-1.8 (-3.0 to -0.6)	-0.27	-1.7 (-3.3 to -0.2)	-0.24
CORE-OM							
Bibliotherapy with support (n = 44)	37.0 (17.7)	24.4 (14.0)	27.2 (17.0)	-12.5 (-17.0 to -8.0)	-0.79	-9.7 (-14.7 to -4.7)	-0.56
Bibliotherapy (n = 45)	36.7 (15.8)	32.5 (16.1)	29.6 (15.4)	-4.2 (-8.5 to 0.1)	-0.26	-7.1 (-11.6 to -2.7)	-0.46
Waiting list control (n = 44)	41.7 (15.6)	36.2 (15.3)	36.0 (18.4)	-5.5 (-8.9 to-2.0)	-0.36	-5.7 (-9.6 to -1.7)	-0.33

Abbreviations: Pre, Pre-treatment assessment; Post, Post-treatment assessment; Fu3, 3-month follow-up assessment; CORE-OM, The Clinical Outcomes in Routine Evaluation-Outcome Measure

^a ^Effect sizes calculated within groups, pre-post, and pre-fu3, with pooled SDs. (A negative effect size represents a decrease in the measure, a positive effect size an increase.)

**Table 7:** Day time functioning and psychological distress; Interactions

	Pre to Post						Pre to Fu3					
	BTS v/s WL		BTS v/s BT		BT v/s WL		BTS v/s WL		BTS v/s BT		BT v/s WL	
	*P*	Effect size (η_p_^2^)^a^	*p*	Effect size (η_p_^2^)^a^	*p*	Effect size (η_p_^2^)^a^	*p*	Effect size (η_p_^2^)^a^	*p*	Effect size (η_p_^2^)^a^	*p*	Effect size (η_p_^2^)^a^
Positive DTR	.001	0.12	.05	0.04	.13	0.03	.005	0.09	.42	0.01	.04	0.05
DTF	< .001	0.21	.002	0.11	.28	0.01	< .001	0.15	.11	0.03	.03	0.06
PSS	.02	0.06	.16	0.02	.40	0.01	.05	0.04	.66	0.00	.10	0.03
CORE-OM	.02	0.06	.01	0.07	.65	0.00	.22	0.02	.45	0.01	.63	0.00

Abbreviations: Pre, Pre-treatment assessment; Post, Post-treatment assessment; Fu3, 3-month follow-up assessment; BTS, Bibliotherapy with support; WL, Waiting list control group; BT, Bibliotherapy; Positive DTR, Positive Day Time Ratings; DTF, Day Time Fatigue; PSS, Perceived Stress Scale; CORE-OM, The Clinical Outcomes in Routine Evaluation-Outcome Measure

^a ^η_p_^2 ^(Eta 2) is interpreted as explained variance in the sample

#### Co-morbidity and outcome

At post-treatment, the correlation between total number of co-morbid problems and ISI change scores was small but significant and negative (ρ(87) = -0.22, p = .040). In other words, larger number of co-morbid problems was associated with slightly lower improvements in insomnia severity. This association was not significant at three-month follow-up (ρ(79) = -0.19, p = .095).

To find out if specific co-morbid conditions affected treatment outcome, ANOVAs were performed for the more prevalent co-morbid conditions (i.e. allergy, acute pain, stress, restless legs, nightmares, snoring, bruxism, high blood pressure, chronic pain, nocturia, tinnitus, depression, and anxiety) with ISI as the dependent variable. To increase stability of measurement for each diagnosis, individuals whose problem could not be clearly verified or ruled out at the assessment interview were not included in these analyses. ANOVAs showed significant main effects of group only for chronic pain (F_(1, 131) _= 6.937, p = .009) and stress (F_(1, 105) _= 6.633, p = .011), i.e. individuals with chronic pain or stress problems suffered more severe insomnia at all occasions. Only individuals with nightmares responded to the treatment with less marked improvements on the ISI, as seen by an interaction effect for nightmares (F_(1.83, 221.27) _= 3.566, p = .034).

#### Sleep medication use

Out of 59 individuals using sleep medication at pre-assessment (see table 1), 21 had ceased sleep medication at the post-assessment interviews. Relatively few of these, 4 out of 25 (16.7%) and 3 out of 14 (21.4%), were found in the waiting list and bibliotherapy groups respectively, compared to 14 out of 20 (70%) in the group receiving bibliotherapy with therapist support. This advantage for the group receiving bibliotherapy with therapist support was significant (χ^2 ^= 15.179, df = 2, p > .001, N = 58).

To control for the possibility that non-users started to use sleep medication during treatment, the total number of participants using sleep medication post-treatment was also compared. In this analysis, 7 were found in the group receiving therapist support, 11 in the bibliotherapy only group, and 21 in the waiting list control group, and this difference was significant (χ^2 ^= 12.181, df = 2, p = .002, N = 132). At three-month follow-up assessment, 7 participants in the group receiving therapist support used sleep medication, which was significantly lower than 15 in the bibliotherapy group, and 19 in the waiting list control group (χ^2 ^= 8.355, df = 2, p = .015, N = 130).

### Adverse events

The most important adverse event was one individual in the treatment group with support who dropped out of treatment due to increased pain as an effect of sleep restriction. In all, 23 individuals in the treatment groups reported one adverse event and 2 individuals reported two adverse events. More specifically, 9 felt that sleep restriction made them more tired or was too demanding, 2 individuals in the treatment group without support dropped out of treatment because some part of the treatment was too demanding, 3 felt the sleep diary increased their sleep related concerns, or was too demanding to fill out, 4 did not agree with the suggested life-style changes or sleep-wake rhythm, another 2 had trouble sleeping when ceasing sleep medication, and 1 experienced increases in other problems when sleep was no longer a problem. The remaining 5 experienced slight adverse experiences, such as having a hard time not watching TV in bed, or not drinking coffee in the evenings, and one reported having problems in that their sleep was so sound after treatment that they did not hear the alarm in the morning.

## Discussion

In this study, we demonstrate that using a self-help book to deliver a CBT-treatment can markedly reduce insomnia severity, and improve sleep and day-time functioning, in adults with insomnia and co-morbid problems. The effects can be enhanced by adding brief, structured weekly therapist support over the telephone.

As hypothesized, the results show that self-help bibliotherapy had a strong positive effect on sleep in this group of participants with insomnia and co-morbid problems. The effects were manifest both for remission rates, sleep timing and subjective measures of sleep as well as for ratings of day-time functioning. Gains seen immediately after treatment were to a large extent maintained three months later.

Therapist supported treatment produced larger overall effects at post-treatment as well as at three-month follow-up, compared to treatment without therapist support or no treatment. The number of participants with clinically significant improvements in insomnia severity (i.e. responders and remitters) was larger, effects on sleep, day-time functioning and perceived stress were larger, and medication use was greatly reduced in the group receiving therapist support as compared to the group not receiving therapist support, and the waiting list control group. Although the difference compared to treatment without support was somewhat attenuated at three-month follow-up, participants in the therapist-supported group were still better off in several important aspects. In contrast, Mimeault and coworkers found the extra effect of brief therapist contact to become negligible at three-month follow-up [29]. However, our findings are in line with the Van Straten & Cuijpers meta-analysis of self-help treatments of insomnia [17], as well as research in self-help for areas such as depression and anxiety, and support the notion that therapist contact with participants does enhance treatment outcome [28].

The effect of therapist support is impressive considering its limited amount (15 minutes per week for 6 weeks) and the fact that the support took place over the telephone. In fact, effects in this group compare well to those demonstrated for primary insomnia, both in a recent study of CBT group-therapy and CBT + medication [38], and in a meta-analysis on CBT and medication [13]. This may be due to the high level of structure of the telephone calls, focusing on sleep restriction and stimulus control. In contrast, bibliotherapy without therapist support produced slightly lower effect sizes for most measures, comparable to those seen previously in self-help treatments for insomnia [17].

Considering that the majority of patients with insomnia also suffer from co-morbid problems [22,46], the present findings of positive treatment effects in insomnia severity in a group with heterogeneous co-morbid problems may be of great importance. It should be noted, however, that larger number of co-morbid problems was associated with slightly lower improvements in insomnia severity. Although only 4% of the variance in outcome was explained by co-morbidity, the findings point at a need to further evaluate the influence of co-morbid disorders. For instance, studies evaluating the effect of CBT for insomnia for specific combinations of insomnia and co-morbid conditions are needed. One possibility is to develop and test combined manuals. For example, in the present study, individuals with nightmares experienced significantly lower gains from treatment, which could indicate a need to improve the treatment for this rather large group. Since specific treatments of nightmares have already been developed (e.g. Imagery Rehearsal Therapy [47]), evaluating the effect of IRT on insomnia symptoms, and combining CBT for insomnia and IRT protocols for this group should be considered for individuals with both insomnia and nightmares. As mentioned earlier, Mimeault and co-workers [29] did not find lasting differences between the groups receiving support and not. However, only participants with primary insomnia were included in that study. The findings in the present study could indicate that individuals with co-morbid disorders may benefit more from therapist support than do individuals with primary insomnia. From a clinical point of view, such a relation would be of great importance and merits further investigation.

Several limitations should be noted in the present study; e.g. the use of self-reports as opposed to objective sleep timing measures. Although sleep diaries represent a core assessment component in insomnia research [48], patients with insomnia generally over-estimate wake-time [49]. Nonetheless, sleep diaries have been shown to correlate well with objective measures [50]. It is likely that these results can be compared to those of other studies using sleep diaries, but objective measures, such as polysomnography or actigraphs, would be needed to confirm objective changes in sleep. Since we were interested in maximizing generalizability, selection criteria were liberal, which yielded a heterogeneous sample. A concern regarding generalization is that our participants had a high education level, and generally a good or at least adequate economic situation. Finally, the power calculation was based on the expected difference in effect between the two treatments and the wait list control group, and it cannot be excluded that the present study was not adequately powered to fully investigate the differences between the two active treatment arms.

The study also has several strengths. We used an untreated group to control for fluctuations over time up until five months after the beginning of treatment, and analyzed interactions to control for changes not due to treatment. We also had a comparably large number of participants in each treatment group, and included measures of day-time functioning, which have not been adequately studied in earlier insomnia treatment research. The heterogeneous sample extends previous positive findings on individuals with primary insomnia to also include the typical insomnia patient with co-morbid problems.

## Conclusions

Self-help CBT-based bibliotherapy can effectively alleviate insomnia in a self-recruited sample with a wide range of co-morbid disorders. Brief structured guidance from a therapist enhanced outcome, and treatment gains as well as differences between groups were largely maintained three months after the end of treatment. In particular when considering its potential for being easily distributed and low in cost, self-help CBT alone or with brief therapist support is likely to help the large group of individuals with insomnia including those with co-morbid problems.

## Competing interests

Financial disclosure: Susanna Jernelöv is the author of the commercially available self-help book used in this study. We are aware of no other competing interests.

## Authors' contributions

SJ contributed to the conception and design of the study, wrote the treatment manual, and conducted supervision of therapists. She also took part in the acquisition, analysis and interpretation of data, and was the main author of the manuscript. All authors contributed to drafting and critical review of the manuscript and approved the final version. In addition, ML participated in conception and design, analysis and interpretation of data. KB and SR contributed to conception and design, acquisition, analysis and interpretation of data, and also acted as therapists. BL participated in acquisition and analysis of data and technical support. JA contributed to the conception and design, and interpretation of data. VK contributed to the conception and design, acquisition, analysis and interpretation of data, and statistical expertise.

## Pre-publication history

The pre-publication history for this paper can be accessed here:

http://www.biomedcentral.com/1471-244X/12/5/prepub
